# Giant Primary Retroperitoneal Teratoma in an Adult: A Case Report

**DOI:** 10.1155/2010/650424

**Published:** 2010-08-24

**Authors:** Poonam Mathur, Miguel A. Lopez-Viego, Myron Howell

**Affiliations:** ^1^College of Osteopathic Medicine, Nova Southeastern University, Davie, FL 33314, USA; ^2^Bethesda Memorial Hospital, Boynton Beach, FL 33435, USA; ^3^University of Miami, Coral Gables, FL 33124, USA; ^4^Florida Atlantic University, Boca Raton, FL 33431, USA

## Abstract

Teratomas are bizarre neoplasms derived from embryonic tissues that are typically found only in the gonadal and sacrococcygeal regions of adults. Retroperitoneal teratomas are rare and present challenging management options. We report here the case of a histologically unusual retroperitoneal tumor detected on computed tomography during the workup of abdominal pain in a 32-year-old male. The evaluation and treatment of this condition and a review of the literature are included in this paper.

## 1. Introduction

Teratomas are tumors that are derived from embryonal tissue and composed of somatic cell types from two or more germ layers (ectoderm, mesoderm or endoderm) [1]. A teratoma is considered to be a non-seminomatous germ cell tumor and is typically located in either the sacrococcygeal region or in the gonads. Malignant mature cystic teratomas (0.2 to 2% of cases) [2] have the potential to metastasize to sites such as the retroperitoneal lymph nodes and lung parenchyma [1]. Retroperitoneal teratomas are rare in adults, typically occurring in this location only in infancy and childhood [3]. We present a rare case of a massive retroperitoneal tumor in a 32-year-old patient, which was treated successfully with surgical resection. A review of the current clinical literature on this topic supports our management of this case.

## 2. Case Presentation

**Figure 1:**
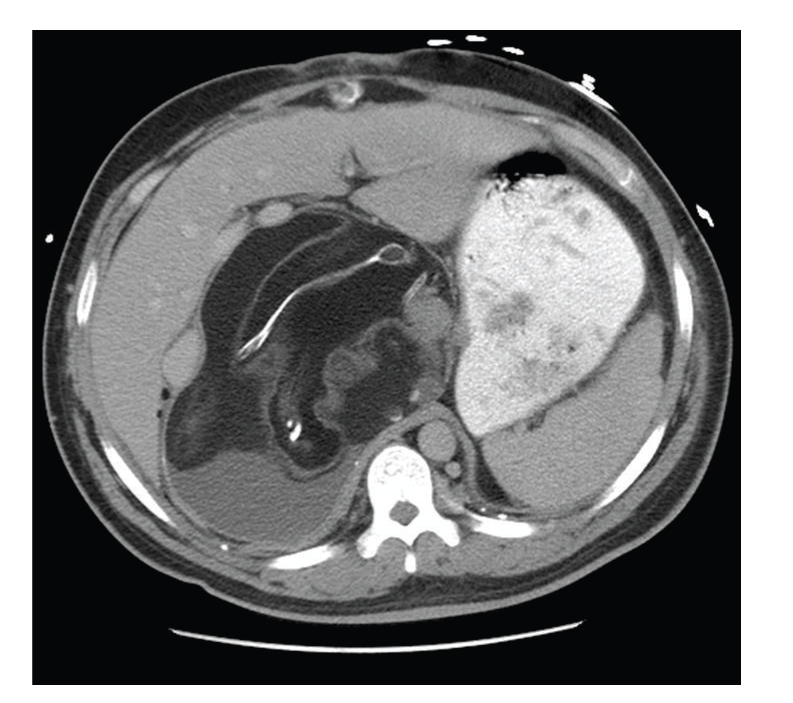
CT scan superior to the umbilicus shows a large, right-sided retroperitoneal mass with bone and soft tissue.

**Figure 2:**
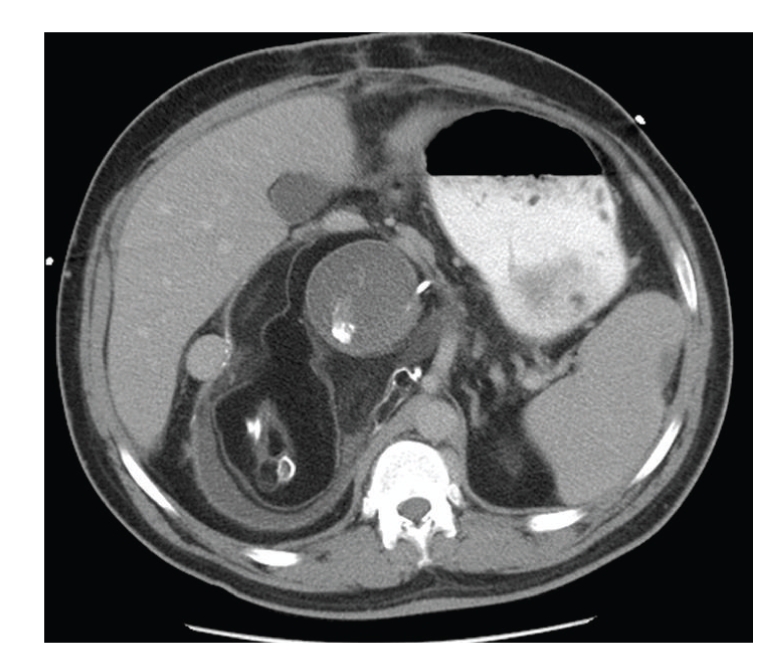
CT scan with contrast shows a retroperitoneal mass containing bone, soft tissue, and cystic elements.

A 32-year-old Hispanic male presented to the emergency department with a complaint of abdominal pain and fullness. The pain was mostly localized to his right lower quadrant and right flank. He was admitted to the hospital where, on computed tomography, he was found to have a large retroperitoneal mass containing bone and multiple different soft-tissue densities consistent with a teratoma (Figures 1 and 2).

The patient's past medical history is significant for adult-onset diabetes, obesity, and hypercholesterolemia. Additionally, in April 2009, he was involved in a motor vehicle accident in Cuba where he underwent an exploratory laparotomy for reported peritonitis from a perforated viscus. Exact details of that operation and its findings were unavailable. The physical examination revealed a well-healed midabdominal surgical scar, abdominal distension, and minimal tenderness in the right flank. A recent barium upper GI study revealed an unremarkable stomach, duodenum, and small bowel series, with a large radiolucent mass in the right upper quadrant, corresponding to the previously diagnosed teratoma. Admission laboratory tests showed no abnormalities.

The patient underwent resection of this lesion through a thoracoabdominal incision. The diaphragm was incised radially and the neoplasm was dissected from its attachments to the right kidney, vena cava, liver, hepatic veins, and diaphragm. The retroperitoneal dissection was tedious and difficult, but the mass was excised in its entirety. The chest was closed with a 36 French thorcostomy tube and the patient recovered without incident. 

**Figure 3:**
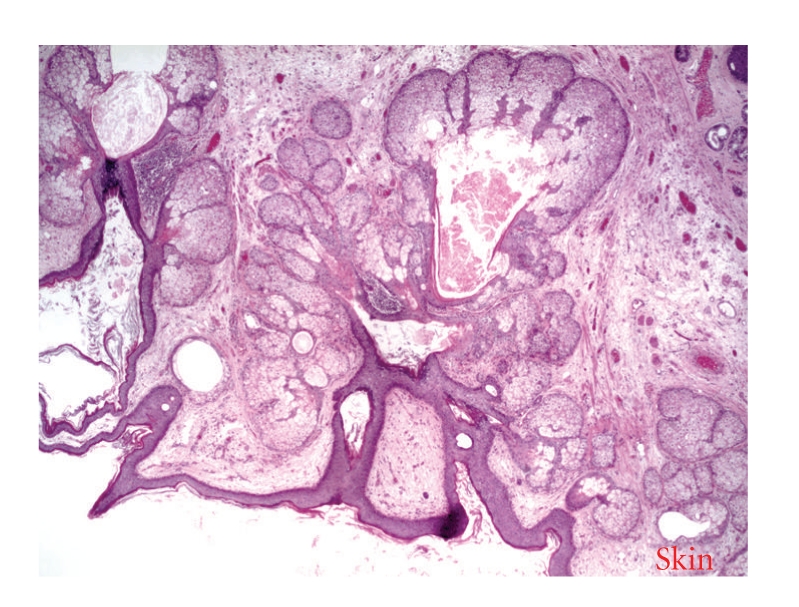
Skin adnexa in teratoma.

**Figure 4:**
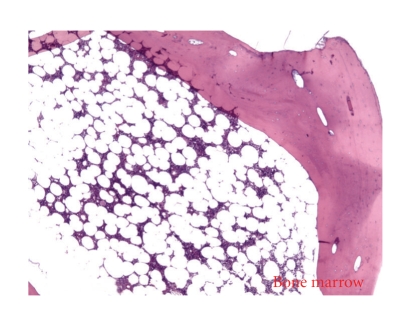
Bone components in teratoma.

**Figure 5:**
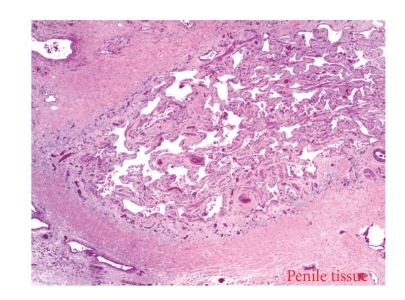
Penile tissue in teratoma.

The resected retroperitoneal mass was found to be a benign extragonadal teratoma, measuring 18.0 × 12.5 × 9.0 cm, partly covered with a smooth, gray-brown membrane. The external surface was lobulated and ragged in areas, with cut sections revealing mostly yellow, lobulated fat with cystic areas filled with brown, cheesy material and hair (Figure 3). Areas of calcification compatible with bone were also noted (Figure 4). Penile tissue was also present microscopically (Figure 5).

## 3. Discussion

Teratomas are the most common type of germ cell tumor in humans, and most of these neoplasms are benign. They are typically classified into three general categories: mature (cystic/solid, benign), immature (malignant), and mondermal (highly specialized) [2]. Each of these histologies may present alone or in combination with others. A mature teratoma consists of an adult-type tumor with well-differentiated elements, while an immature teratoma consists of elements with only partial somatic differentiation, similar to those seen in embryonic or fetal tissue [4]. Teratomas often contain dermal elements and can also be classified by the type of epithelium and dermal tissue contained within the tumor. Epidermoid teratomas contain stratified squamous epithelium; dermoid teratomas may contain any kind of epithelium and dermal elements such as hair and glands, and teratoid teratomas are lined with any respiratory epithelium (usually it is columnar epithelium) and contain sebum [1]. It is important to note that teratomas are always considered foreign to the anatomical region in which they are found despite their tendency to differentiate into somatic-type tissues. It is thought that these cysts may develop by failure of meiosis I or from a premeiotic cell in which meiosis I has failed [2]. There is no specific tumor marker for teratomas though some immature (malignant) teratomas have been associated with elevated alpha-fetoprotein (AFP) levels [5].

Retroperitoneal teratomas are rare entities, representing only 1%–11% of all primary retroperitoneal tumors. Incidence is bimodal with peaks in the first 6 months of life and in early adulthood. Due to their location, they are usually identified only after they have grown to huge proportions [6]. Additionally, the incidence of retroperitoneal teratomas in females is twice that in males. When retroperitoneal teratomas do occur, they are often located near the upper pole of the kidney, with preponderance on the left side. Although these tumors are mostly asymptomatic, they can cause abdominal distension and pain, as well as nausea and vomiting via compression of surrounding structures [3]. The diagnosis of retroperitoneal teratoma often can be made on the basis of radiologic imaging. Retroperitoneal teratomas can be predominantly cystic or completely solid in appearance. A computed tomography (CT) scan or magnetic resonance image (MRI) can identify various components of these tumors, including bone, soft-tissue density structures, adipose tissue, and sebaceous and serous-type fluids. These imaging studies also can display the precise location, morphology, and adjacent structures of the tumor, which provide better preoperative planning and increased likelihood of complete removal of the tumor with less iatrogenic damage [4].

Surgical resection remains the mainstay of therapy for mature teratomas and is required for definitive diagnosis [6]. Resectability is determined by pathologic category and extent of tumor. Resection can include segments of the GI tract, kidney, bladder, spleen, aorta, and vena cava [7]. Torsion is common with these tumors, and if rupture occurs, sebaceous material can spill into the abdominal cavity, causing shock, hemorrhage, or a marked granulomatous reaction that can lead to adhesion formation [2]. An imaging study is critical for developing the preoperative strategy and performing a safe surgical excision.

Benign tumors, when resected, yield a 5-year survival rate of 100%. A long-term study showed that complete surgical resection is associated with the best survival rates for primary retroperitoneal tumors [7]. Disease-free survival following resection of teratoma is related to completeness of resection; therefore, there are significant advantages to surgery with low volume disease. Moreover, there is the risk of malignant transformation of teratoma to carcinoma or sarcoma, so unresected teratoma may result in late relapse (defined as recurrence after a relapse-free interval of more than two years after completion of primary treatment). A late relapse often shows a slow growth and usually responds poorly to chemotherapy [8].

Testicular ultrasound is necessary to rule out a coexisting testicular germ cell tumor in male patients. This is a necessary step since 50% of men with retroperitoneal tumors also have testicular carcinoma in situ, a precursor for testicular germ cell tumors [5]. Long-term care also involves advising patient to follow up with annual CT to detect relapse at an asymptomatic phase [8].

## 4. Conclusion

Primary retroperitoneal teratoma is a rare entity in adults. Although usually asymptomatic, large neoplasms can cause abdominal and flank pain. Preoperatively, the diagnosis can be established by its characteristic appearance on computed tomography. The definitive treatment for these neoplasms is surgical resection.
